# Management of women with endometriosis in the 21st century

**DOI:** 10.1097/GCO.0000000000001027

**Published:** 2025-04-10

**Authors:** Attilio Di Spiezio Sardo, Christian M. Becker, Stefan P. Renner, Pia A. Suvitie, Josep Estadella Tarriel, Silvia Vannuccini, Juan A Garcia Velasco, Jasper Verguts, Antonio Mercorio

**Affiliations:** a Department of Public Health, School of Medicine, University of Naples Federico II, Naples, Italy; b Endometriosis CaRe Centre, Nuffield Department of Women’s and Reproductive Health, University of Oxford, Oxford, United Kingdom; c Department of Gynecology and Obstetrics, Hospital Böblingen, Klinikverbund-Suedwest, Klinikum Sindelfingen-Böblingen, Böblingen, Germany; d Department of Obstetrics and Gynecology, Turku University Hospital and University of Turku, Turku, Finland; e Obstetrics & Gynecology Department, Hospital Universitari de la Santa Creu i Sant Pau, Pediatrics, Obstetrics and Gynecology, and Preventive Medicine and Public Health Department, Universitat Autònoma de Barcelona, Barcelona, Spain; f Department of Experimental and Clinical Biomedical Sciences ‘Mario Serio’, University of Florence, Obstetrics and Gynecology, Careggi University Hospital, Florence, Italy; g IVIRMA Global Research Alliance, IVI RMA Madrid, Instituto de Investigación Sanitaria La Fe (IIS La Fe), Valencia,Rey Juan Carlos University, Madrid, Spain; h Department of Obstetrics and Gynaecology, Jessa Hospital, Faculty of Medicine, Hasselt University, Hasselt, Belgium; i Department of Obstetrics, Gynecology and Reproductive Medicine, Hospital Foch, Suresnes and University Versailles, Saint-Quentin en Yvelines, France.

**Keywords:** endometriosis, infertility, minimally invasive surgery, pelvic pain

## Abstract

**Purpose of review:**

Endometriosis is a chronic inflammatory condition that significantly affects women’s quality of life and fertility. Despite advancements in treatment, many areas of uncertainty persist in clinical management. This review provides a symptom-focused, patient-centered update, addressing cases from asymptomatic to those complicated by pain and infertility

**Recent findings:**

Advancement in imaging technology has increased incidental diagnoses of asymptomatic endometriosis, raising the debate between immediate treatment and watchful waiting. Medical therapy primarily aims to suppress symptoms, with oral gonadotropin-releasing hormone antagonists and add-back therapy offering promising long-term pain control. Research into local neurogenesis and central sensitization supports complementary approaches, though high-quality evidence is still limited. For pain refractory to medical therapy, conservative surgical strategies can minimize postoperative complications without significantly increasing recurrence rates. In infertility, assisted reproductive technology (ART) provides effective options, although the optimal endometrial preparation and the necessity of pre-ART surgery remain to be fully elucidated

**Summary:**

The optimal management of endometriosis requires a personalized, multidisciplinary approach within specialized centers. Long-term suppressive medical therapy remains the cornerstone of pain management while emerging targeted agents hold promise for better symptom control with fewer side effects. Surgical intervention should be performed by experienced surgeons as a single definitive procedure when possible. Tailored ART protocols can address infertility challenges. Standardized classification systems and robust randomized trials are crucial to refining treatment pathways, optimizing fertility outcomes, and enhancing quality of life.

**KEY POINTS**

- Asymptomatic endometriosis is increasingly identified through advanced imaging, raising debate about initiating medical therapy or opting for close surveillance in the absence of symptoms.
- New oral GnRH antagonists, combined with ABT, achieve sustained, long-term pain relief and acceptable bone safety, representing a promising second-line treatment.
- Multidisciplinary care, ideally in specialized centers, integrates conservative surgery, fertility preservation, and assisted reproduction, emphasizing a single definitive intervention to optimize outcomes.

## INTRODUCTION

Endometriosis is a chronic inflammatory disease characterized by endometrial-like tissue growing outside the uterus, commonly associated with pelvic pain and infertility [1]. It affects approximately 2–10% of the general population, nearly 40% of infertile women [2], and over 60% of those with chronic pelvic pain [3].

The classification of endometriosis is contentious and complex, as existing systems mainly focus on lesion size [4]. These classifications are criticized for weak correlation with symptom severity and inability to predict prognosis, leading to inconsistent clinical management often influenced by physicians’ beliefs and local practices rather than robust evidence from randomized trials [5]. This highlights the need for integrative systems aligned with clinical outcomes to support standardized, evidence-based care.

Dysmenorrhea and infertility, the principal symptoms of endometriosis, exert a profound negative impact on patients’ quality of life (QoL), social interactions, and sexual well-being [6].

Therefore, effective management should begin by addressing these core symptoms – pain and infertility – through treatments tailored to each patient’s age, ovarian reserve, and reproductive goals.

In this review, we examine the latest literature to define an updated, symptom-focused management strategy that prioritizes the patient’s needs, placing her experience at the center of care planning.

## MANAGEMENT OF ASYMPTOMATIC ENDOMETRIOSIS

With advancements in imaging quality – particularly transvaginal ultrasound – and the introduction of more noninvasive techniques such as the salivary miRNA test, an increasing number of asymptomatic patients are incidentally diagnosed with endometriosis, often with ovarian endometriomas, despite the absence of pelvic pain or infertility [7]. This raises the question of whether these patients should undergo treatment or simply be monitored through regular follow-up.

The progression and evolution of endometriosis remain unpredictable, making it essential for clinical management of asymptomatic patients to carefully balance the risks of intervention against the potential benefits of surveillance.

According to the European Society of Human Reproduction and Embryology (ESHRE) guidelines, clinicians are advised against using peripheral biomarkers, such as CA125, for routine serial monitoring of endometriosis. Regular serum biomarker level determinations have not been shown to consistently enhance endometriosis management and instead tend to increase costs and the overall treatment burden for patients [8,9].

In this context of asymptomatic endometriosis, the decision to initiate treatment remains highly uncertain. Theoretically, creating a hypoestrogenic environment by reducing ovarian estrogen levels and blocking menstrual cycle secretion through inhibition of the hypothalamic-pituitary-ovarian axis aligns logically with the physiopathogenesis of endometriosis, making it a seemingly effective strategy to prevent disease progression. There is, however, currently no follow-up data in the literature that definitively supports the efficacy of this approach in halting endometriosis evolution.

Theoretically, the progesterone-only pill, the most studied molecule for endometriosis due to its safety profile, acceptable tolerability, and low cost [10], is to be preferred. In the absence of clear therapeutic superiority, treatment, however, can be tailored according to patient compliance, with the combined oral contraceptive (COC) pill serving as a valid alternative [11].

## FERTILITY PRESERVATION

Whenever ovarian surgery is indicated, fertility preservation (oocyte cryopreservation) has increasingly emerged as a well-established strategy. Indeed, a significant decrease in anti-Müllerian hormone levels is observed more frequently following cystectomies for endometriosis compared with other benign ovarian conditions. This is because endometriomas are ‘pseudocysts’, lacking a well-defined cleavage plane; thus, the commonly used stripping technique during cystectomy often results in the inadvertent removal of healthy ovarian tissue, compromising the ovarian reserve [12].

An unequivocal question now arises: for which patients with endometriosis, who are neither candidates for surgery nor currently seeking pregnancy, should fertility preservation still be considered?

The impact of the endometriotic disease itself, and the presence of ovarian endometriomas on follicular reserve independent of any surgical intervention, remains controversial.

Ovarian endometriomas can have detrimental effects on ovarian reserve by mechanically stretching the ovarian cortex and causing inflammatory-mediated damage [13,14]. These effects are evident in histologic evaluation of affected ovaries, which demonstrate reduced follicular density, increased atresia, and increased primordial follicle activation compared with an unaffected ovary [14,15]. From a clinical perspective, Somigliana *et al*. [16] demonstrated a reduced ovarian response to stimulation in the ovary affected by an endometrioma compared with the contralateral, healthy ovary.

Regarding oocyte quality, although increased levels of free iron, reactive oxygen species (ROS), proteolytic enzymes, and inflammatory molecules have been detected in follicles adjacent to ovarian endometriomas, large studies at the moment do not seem to demonstrate a significant impact on oocyte quality [17]. The heterogeneity of patient cohorts and the use of indirect outcomes are limitations that, nonetheless, should always be taken into consideration.

A systematic approach to fertility preservation through oocyte cryopreservation should be considered for patients with ovarian involvement at risk of a quantitative reduction in follicular reserve. This includes cases of recurrent endometriomas, multiple surgeries, bilateral endometriomas >3 cm, or large unilateral endometriomas (≥5 cm) [18]. This approach is particularly beneficial for younger patients (<35 years), as they are likely to gain the maximum advantage from oocyte cryopreservation.

Based on the patient's age and the estimated number of oocytes required to achieve a live birth, 1–3 cycles of ovarian stimulation may be performed to accumulate oocytes before and/or after surgery, depending on endometrioma size [19].

In an observational study of 1044 women, Cobo *et al*. [20] reported that 46% eventually utilized their cryopreserved oocytes. Fertility preservation is currently not recommended for patients who do not present with endometriomas and have a normal ovarian reserve [21].

## ADDRESSING PAIN IN ENDOMETRIOSIS

### Hormonal treatment

All currently available treatments for endometriosis are suppressive, not curative. Upon discontinuation, symptom recurrence is the rule [22].

All guidelines recommend long-term hormonal treatment to reduce estrogen production, as it is proinflammatory and responsible for the proliferation of endometriotic lesions [23]. No single therapy has demonstrated clear superiority. Decisions regarding hormonal treatment should consider patient preferences and tolerability [24].

In the latest ESHRE guidelines, dienogest (DNG) is recommended as a first-line treatment option [8]. When administered continuously, it induces decidualization of endometrial tissue, leading to subsequent atrophy of endometriotic lesions [25].

A pooled analysis of four clinical studies confirmed that DNG 2 mg has a strong safety profile for up to 65 weeks [26], with mild to moderate adverse events – such as headache, breast discomfort, depressed mood, and acne – affecting less than 10% of patients and resulting in low discontinuation rates. Extended use for up to 5 years also demonstrated favorable safety outcomes [27,28]. It should, however, be noted that progestin-only pills or COCs are effective in managing endometriosis-related pain in approximately two-thirds of affected women [29]. The concept of progesterone resistance provides insight into why about one-third of patients do not respond to these treatments [30].

Oral nonpeptide gonadotropin-releasing hormone (GnRH) receptor antagonists represent a promising treatment for endometriosis-associated pain when first-line options have failed, offering efficacy comparable to GnRH agonists with advantages such as oral administration, rapid onset, no flare-up effect, and reversible, dose-dependent suppression of ovarian steroid secretion. By blocking GnRH receptors in the anterior pituitary, they reduce luteinizing hormone and follicle stimulating hormone secretion, leading to lower estradiol and progesterone levels and inducing a hypoestrogenic state. This state, however, may cause undesired effects like vasomotor symptoms onset and bone mineral density (BMD) decline, requiring careful monitoring and restricted treatment duration [31].

Elagolix is approved in the USA for reducing endometriosis-associated pain (Orilissa USPI, 2023). The use of its highest dose (200 mg twice daily), however, is limited to 6 months due to concerns about BMD loss. The lower dose (150 mg daily) is approved for use for up to 2 years, though the dysmenorrhea response rates are not as robust as in the group receiving the higher 200 mg dose twice daily. Additionally, its impact on dyspareunia and the need for rescue analgesics did not show a statistically significant difference compared with placebo [32].

Relugolix combination therapy (Relugolix CT) enhances compliance by integrating add-back therapy (ABT) into a single pill, allowing for long-term treatment with minimal impact on bone mass. In the 24-week study to prospectively investigate the relugolix combination therapy in the management of endometrial-associated threatening pain (SPIRIT) pivotal trial and the subsequent 80-week long-term extension (LTE) studies, Relugolix CT (40 mg relugolix, 1 mg estradiol, and 0.5 mg norethisterone acetate) demonstrated sustained improvement in endometriosis-associated symptoms and a reduction in analgesic use, including opioids [33].

In the SPIRIT LTE study on QoL, measured using the Endometriosis Health Profile-30 questionnaire, Relugolix CT treatment for up to 2 years showed rapid and sustained improvements in various aspects of QoL. Notably, despite an initial BMD decrease of less than 1% at the most estrogen-sensitive site – the lumbar spine – mean BMD remained stable over time [34^■^]. This aligns with the estrogen threshold hypothesis, which identifies a therapeutically effective estradiol range (30–45 pg/ml) that promotes improvement in endometriosis symptoms while minimizing hypoestrogenic side effects [35].

Clinical data are promising, indicating that Relugolix CT could be a valuable option for addressing the need for safe, effective, and well-tolerated long-term treatments for endometriosis. It may reduce opioid reliance and improve patients’ QoL.

When treating endometriosis with medical therapy, recognizing the biochemical heterogeneity of lesions is essential. Altered expression of key enzymes, such as variable aromatase activity and progesterone resistance, may explain why therapy is effective for about 70% of women but has limited or no effect in 10 and 20% of cases, respectively. This variability highlights the need for new drugs targeting local estrogenic activity while minimizing effects on gonadal function. Due to the complexity of enzyme expression evaluation, many aspects, however, remain unclear [36].

### Alternative and complementary approaches

Interdisciplinary evaluation by gynecologists, pain specialists, sexologists, psychologists, physiotherapists, and social workers is essential to ensure comprehensive care for endometriosis patients [37].

Central sensitization is increasingly recognized as a critical factor in the development of endometriosis-related pain, amplifying signals from peripheral sources [38]. This phenomenon is linked to myofascial trigger points and associated psychological comorbidities [39]. Additionally, endometriosis-related local neurogenesis may further intensify pain. Research involving patients with cul-de-sac or uterosacral endometriosis, particularly those experiencing deep dyspareunia, has shown a significantly higher density of nerve bundles in these individuals [40], likely influenced by nerve growth factor – a key neurotrophic factor in endometriosis [41].

Further investigation into the mechanisms driving local neurogenesis in endometriosis is needed to explore new therapeutic avenues.

An online national survey in Australia found that 60–70% of people with endometriosis-associated pelvic pain use nonmedical management strategies, such as acupuncture, Chinese herbal medicine, physiotherapy, exercise, and nutritional interventions [24]. Engaging in these approaches can help individuals regain a sense of control over their conditions. The recommendation for these treatments, however, remains limited due to a lack of high-quality evidence on their effectiveness.

A notable pilot study, the first to compare the effect of low FODMAP (fermentable oligosaccharides, disaccharides, monosaccharides and polyols) diet and the endometriosis diet (developed by women diagnosed with endometriosis and based on the avoidance of nutrients, e.g. red meat, caffeine, sugar) on endometriosis-related symptoms and QoL, suggest that both dietary interventions could reduce both cyclical and noncyclical symptoms, including gastrointestinal issues [42].

### Surgical treatment

When pain persists despite medical therapy, surgery has demonstrated effective outcomes, particularly in cases of deep infiltrating endometriosis (DIE).

A large multicenter observational study by the British Society for Gynaecological Endoscopy involving nearly 5000 cases demonstrated that laparoscopic surgery for rectovaginal endometriosis significantly improved QoL at 6 months and 2 years postsurgery, with reduced opioid use [43].

In a prospective longitudinal study by Bafort *et al*. [44] on 125 patients who underwent complete laparoscopic excision, the comparison of the presurgery and postsurgery evaluation showed significant reductions in dyspareunia, particularly in cases of rectovaginal involvement. These findings suggest that deep dyspareunia in the presence of rectovaginal endometriosis, even as an isolated symptom, is a valid indication for surgery.

Deep endometriosis, though benign, can mimic malignancy due to its invasive nature, requiring complex surgeries best performed in specialized centers. Preoperative evaluation of disease extent and alignment of surgical goals with patient expectations is crucial. Radical surgery carries significant risks, including rectovaginal fistulas, anastomotic leakage, stenosis, and voiding dysfunction. Accurate preoperative counseling, supported by thorough imaging and clinical assessment, is essential to inform patients about potential symptom persistence and complication risks [45].

The future challenge lies in identifying the optimal timing for surgery, ideally achieving a single procedure over a patient’s lifetime with endometriosis [46]. Evidence shows that multiple laparoscopic surgeries are associated with significantly poorer health-related QoL [47].

The principle of complete excision in endometriosis surgery, including bowel resections with safety margins, is being re-evaluated due to evidence of microscopic endometriosis nests distant from nodules, contributing to similar recurrence rates between bowel resections and conservative excisions. Additionally, a growing understanding of the sympathetic nervous system has led to more conservative surgeries to minimize functional complications [48]

**FIG. 1.**
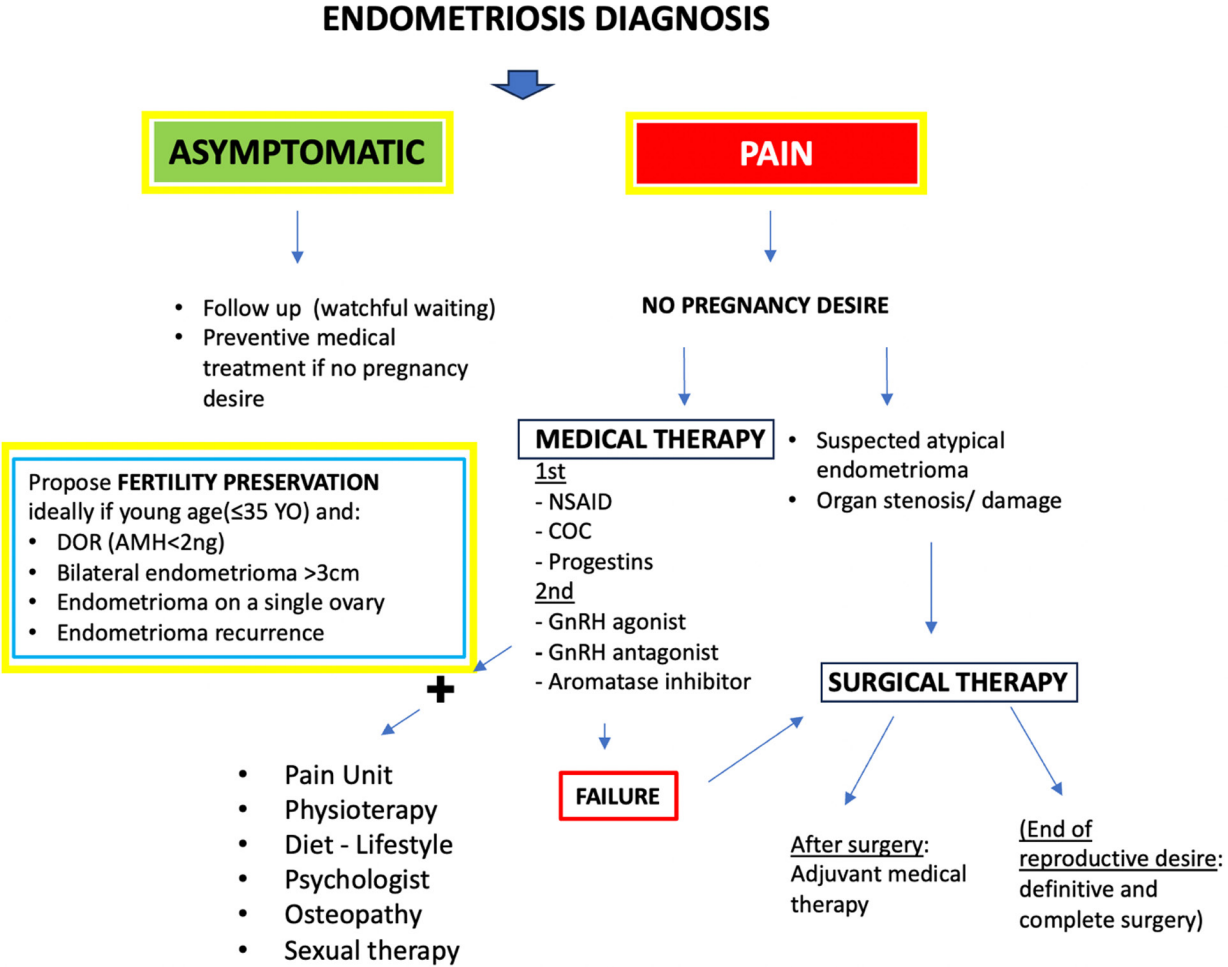
Management strategies for endometriosis in noninfertile women with or without pain. AMH, anti-Müllerian hormone; COC, combined oral contraceptive; DOR, diminished ovarian reserve; GnRH, gonadotropin-releasing hormone; NSAID, nonsteroidal anti-inflammatory drug.

A recent meta-analysis of 13 studies (nonrandomized) on bowel function in women with DIE found that conservative approaches, such as shaving or discoid excision, result in fewer instances of constipation and frequent bowel movements compared with colorectal segmental resection. These findings highlight the need to carefully balance the extent of surgical radicality against potential side effects [49] (Fig. 1).

## APPROACH TO INFERTILITY IN ENDOMETRIOSIS

The association between endometriosis and infertility is well documented, with a prevalence of endometriosis in 40% of infertile women compared with 10% in the general reproductive-age population [50]. Chronic pelvic inflammation disrupts conception by causing adhesions that alter pelvic anatomy and impair oocyte release and transport. Furthermore, elevated levels of cytokines, growth factors, prostaglandins, and ROS could affect ovulation, sperm function, fertilization, and embryo migration.

The effectiveness of intrauterine insemination (IUI) for endometriosis-related infertility is uncertain. IUI may improve fertility in women with stage I–II endometriosis, particularly when anatomical distortion is minimal or sexual activity is challenging [51]. It, however, should be cautiously considered, especially for patients with prolonged infertility, diminished ovarian reserve, or those over 35, as it may delay more effective treatments. IVF is a more effective option, helping to address challenges linked to endometriosis-related infertility. Women with endometriosis, however, often face additional difficulties during ART cycles due to ovarian impairment and altered endometrial receptivity [52].

The pathophysiology of ovarian damage, particularly with endometriomas, is well documented [53]. ROS and proteolytic substances infiltrate surrounding tissue, replacing normal ovarian cortical tissue with fibrous tissue and reducing cortex-specific stroma. This fibrosis causes follicular loss and intraovarian vascular injury, leading to poor ovarian response and fewer retrieved oocytes [54]. Additionally, altered oocyte competence has been hypothesized [55].

Molecular abnormalities in the eutopic endometrium of women with endometriosis may impair endometrial receptivity, potentially affecting embryo implantation [56]. Perspectives on these issues, however, remain conflicting, likely due to the heterogeneity of endometriosis and the difficulty in accurately staging the disease, particularly in infertility cases where surgery is often omitted.

Horton *et al*. [57], in a review of 29 studies, reported a 15% reduction in the likelihood of conception after IVF/intracytoplasmic sperm injection (ICSI) in women with endometriosis compared with those without the condition, alongside a 12% lower live birth rate (LBR) in patients with stage III–IV endometriosis. Conversely, a recent meta-analysis by Qu *et al*. [58] found no significant difference in outcomes compared with controls. Notably, aneuploidy rates in endometriosis patients are comparable to those in age-matched controls [59^■^].

Particular attention has been given to endometrial receptivity in endometriosis. A freeze-all strategy, especially for patients with adenomyosis, is logical, as ovarian stimulation in IVF results in supraphysiological levels of steroids, which may further impair endometrial receptivity in these patients compared with controls [60,61]. This can be mitigated by reducing estradiol levels through long-term pituitary downregulation with GnRHa before frozen embryo transfer (FET) [62]. Evidence regarding the benefits of GnRHa downregulation for endometrial preparation in endometriosis patients, however, remains inconclusive [63].

A recent retrospective cohort study of 1413 women with endometriosis compared endometrial preparation regimens for FET, including natural cycles and HRT with or without GnRHa pretreatment (triptorelin or leuprorelin, 3.75 mg) [64]. The study found no protocol to be superior, with GnRHa pretreatment having no impact on LBRs, clinical pregnancy rates, or miscarriage rates. The lack of observed benefits may be due to the 1-month GnRHa therapy duration, as previous studies showing advantages used 3–6 months of treatment [65]

Randomized controlled trials (RCTs) are needed to clarify the impact of various endometrial preparation protocols in endometriosis patients.

Increasing exogenous progesterone for luteal phase support has been proposed to enhance implantation outcomes. Although fundamental evidence suggests progesterone resistance in endometriosis [66], its clinical impact in HRT-FET cycles appears minimal. An observational cohort study of 1784 patients found no significant differences in serum progesterone levels on the day of HRT-FET between women who conceived, regardless of endometriosis status. Therefore, higher progesterone levels do not seem necessary for women with endometriosis undergoing frozen blastocyst transfer [67].

A theoretical concern exists about ovarian stimulation potentially worsening endometriosis-related pain and increasing recurrence rates. Current evidence, however, suggests that ovarian stimulation during IVF does not exacerbate endometriosis symptoms, accelerate disease progression, or raise recurrence rates [68,69].

## MANAGING COEXISTING PAIN AND INFERTILITY

The most effective treatments for endometriosis-associated pain are contraceptive-based, posing a challenge for patients with pain who also wish to conceive. Surgery, in addition to alleviating pelvic pain unresponsive to medical therapy, can improve the chances of natural conception [70]. This dual-benefit approach is particularly advantageous for women with factors such as younger age, adequate ovarian reserve, a short duration of infertility, good-quality sperm, spontaneous ovulation, and patent tubes [71].

Given that, if surgery is performed, couples should be given 6–12 months to attempt natural conception before considering IVF if infertility persists [72]. The benefit of pre-ART surgery remains debated. Some studies suggest that removing endometriosis lesions may improve IVF outcomes [73–75], while others caution that surgery could negatively impact results and expose young women to significant postoperative complications [76,77].

A recent meta-analysis, which did not focus on a specific endometriosis phenotype and used LBR per cycle as the primary outcome, found no significant effect of surgery on IVF/ICSI LBRs [78^■^].

A single meta-analysis has suggested a potential benefit of surgery for DIE on ART outcomes, specifically IVF/ICSI pregnancy rates [79]. The primary outcome, however, was pregnancy rate, not LBR, and discrepancies exist between the meta-analysis and one of its included studies, particularly regarding clinical pregnancies and live births. Moreover, the lack of proven surgical benefits for IVF outcomes must be weighed against the significant morbidity associated with these procedures.

**FIG. 2.**
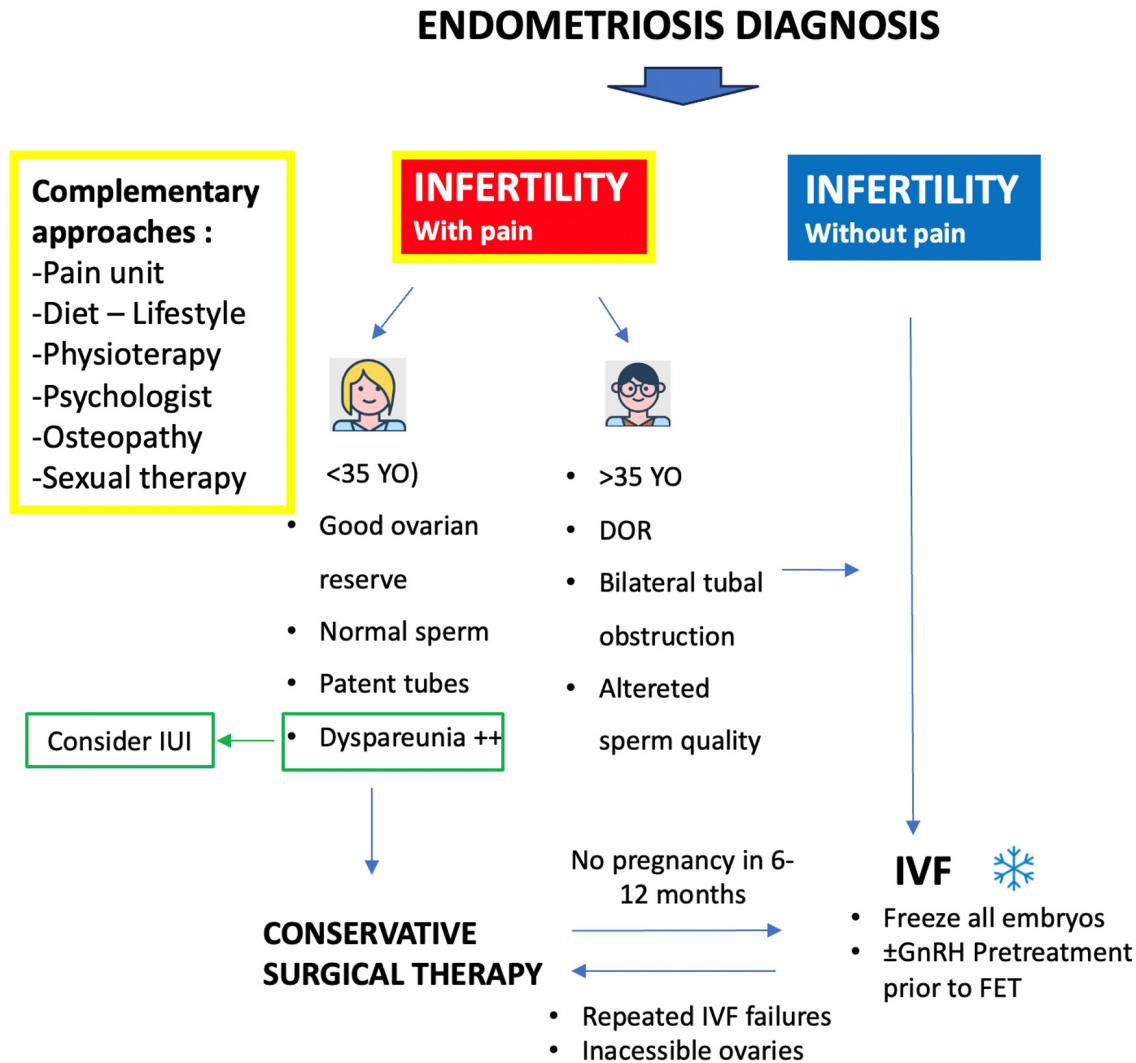
Management strategies for endometriosis in infertile women with or without pain. DOR, diminished ovarian reserve; FET, frozen embryo transfer; GnRH, gonadotropin-releasing hormone; IIU, intrauterine insemination; YO, years old.

Therefore, based on current evidence, endometriosis surgery before IVF should not be recommended to enhance ART outcomes. Surgery, however, may be considered in specific clinical situations, such as for managing uncontrolled pain or in cases of repeated IVF implantation failures [80] (Fig. 2).

## CONCLUSIONS

Effective management of endometriosis in the 21st century requires a patient-centered approach that holistically addresses both their symptoms and overall well-being. This approach requires a personalized, multidisciplinary plan delivered through highly specialized centers.

Endometriosis exhibits a highly variable phenotype, complicating patient comparisons and research advancements. Rigorous RCTs using standardized classification methods are essential for clarifying multiple aspects of disease management.

A diverse array of new medical treatments that target different biological pathways to optimize pain management are expected in the next years. In addition, further research is needed into areas such as local neurogenesis, central sensitization, and the genetic underpinnings of the disease.

When surgery is warranted, it should ideally be a single, definitive procedure performed by an experienced surgeon. This should follow a comprehensive evaluation of the patient to discuss prognosis, set realistic expectations, and review potential risks.

ART has proven effective for treating infertility related to endometriosis; however, further optimization of protocols and deeper insights into the mechanisms behind endometriosis-related infertility are crucial to further enhance fertility outcomes.

## Acknowledgements

*None.*

## Financial support and sponsorship

*Editorial assistance and authors meetings were funded by Gedeon Richter. The sponsor had no role in the preparation or review of the manuscript.*

## Conflicts of interest

*All authors received consultancy fees from Gedeon Richter for the meetings held in preparation for drafting the manuscript.*
